# Increasing incidence associated with herpes zoster infection in British Columbia, Canada

**DOI:** 10.1186/s12879-016-1898-z

**Published:** 2016-10-20

**Authors:** Fawziah Marra, Mei Chong, Mehdi Najafzadeh

**Affiliations:** 1 University of British Columbia, Vancouver, BC Canada; 2 JC2 Answers, Vancouver, BC Canada; 3 Brigham and Women’s Hospital, Harvard Medical School, Boston, USA; 4 University of British Columbia, 2405 Wesbrook Mall, Vancouver, British Columbia V6T 1Z3 Canada

**Keywords:** Herpes zoster, Incidence, Post-herpetic neuralgia, Epidemiology

## Abstract

**Background:**

Recent studies have shown an increasing incidence of herpes zoster (HZ) infection, which may be related to the introduction of varicella vaccination programs in children. We examined the epidemiology and treatment costs of HZ and post-herpetic neuralgia (PHN) over time in British Columbia, Canada.

**Methods:**

The cohort consisted of all cases with HZ infection from January 1, 1997 and December 31, 2012. Incident zoster was defined as a case (ICD-9 053 or ICD-10 B02) without a previous episode of HZ or PHN in the previous 12 months. We determined the incidence for HZ and PHN and the age-sex standardized rate for the overall population. We determined the association between the varicella vaccination program and increased HZ rates by evaluating the rate ratios in the publicly-funded varicella vaccine period compared to the non-publicly funded period in a regression model. We evaluated the hospitalization rates, treatment by GPs and their associated yearly costs for HZ and PHN.

**Results:**

HZ incidence increased for the entire study period from 3.2 per 1000 population in 1997 to 4.5 in 2012. HZ rates were higher for females than males and all age groups had an increased incidence rate, except the 0–9 year olds, where the rate decreased. Crude and age-sex standardized incidence rates of PHN demonstrated very similar patterns to HZ incidence. Based on the regression model, rates of HZ were higher in the older individuals. No significant increase with HZ incidence was seen during the publically funded varicella vaccination program compared to the non-publicly funded period. From 1997 to 2012, the annual HZ-related costs associated with hospitalizations and GP visits were over $CDN4.9 million and $CDN537,286, respectively; treatment costs for hospitalizations have increased significantly over time. Majority of PHN-related cases are managed by GPs, with a steady increase over time in number of cases and associated annual costs.

**Conclusions:**

The incidence of zoster and PHN is increasing with time, particularly in the elderly population and the risk is greater in the over 65 year olds. Treatment costs for both HZ and PHN represent a significant burden on the Canadian healthcare system.

**Electronic supplementary material:**

The online version of this article (doi:10.1186/s12879-016-1898-z) contains supplementary material, which is available to authorized users.

## Background

Varicella zoster virus (VZV) infection or chickenpox typically occurs in children and manifests as a diffuse itchy rash. The rates of infection are high with approximately 95 % of the population testing seropositive for VZV [1, 2]. Reactivation of the varicella virus later in life is known as herpes zoster (HZ) infection [3], and is triggered when an individual’s immune system, cellular immunity in particular, decreases due to the process of aging [4] or becomes immunocompromised [5]. Other risk factors for development of HZ include race (Caucasians are at higher risk than African Americans), gender (most studies show a higher incidence among women), stress, trauma, and diabetes [6–8]. Herpes zoster infection manifests itself as a unilateral, vesicular, painful rash [9], and can lead to complications such as post-herpetic neuralgia (PHN) [10, 11] in approximately 20 % of people.

Recent studies from Europe [12] and North America [13] have shown a trend of increased zoster infection rates over time. Scientists are unclear as to the reason for its increase, but Hope-Simpson in his original studies had hypothesized that viral latency after infection with VZV is maintained by “immunosurveillance”, which is boosted by periodic subclinical reactivations and exposure to exogenous virus [3, 14, 15]. Thus, HZ clinically manifests itself when “immunosurveillance” falls below a certain threshold [3]. Some have hypothesized that the introduction of widespread childhood varicella vaccination programs could lead to less wild type virus boosting for adults [16–18], leading to an increase in zoster rates. Alternatively, the aging population and presence of larger numbers of immunocompromised individuals (due to autoimmune diseases, transplants and larger numbers on medications such as corticosteroids, DMARDS and biologics) [19] could also account for the increase in zoster rates seen around the world [12, 13].

Given that it has been a decade since Canada evaluated its rates of zoster infection [20, 21], we undertook this study to determine the trends over time in age and sex-specific herpes zoster and PHN rates. We further examined the rates of hospitalization and costs associated with HZ and PHN over time. Finally, although not our primary objective, we looked at the impact of varicella vaccination on herpes zoster rates.

## Methods

### Data source

We used population-based data available from PopulationDataBC® which houses several health-related databases, including the Medical Services Plan (MSP) [22] and Discharge Abstract Database (DAD) [23]. These databases use the International Classification of Diseases, Ninth Revision (ICD-9) or Tenth Revision (ICD-10) to code for medical billing. These two databases were linked to the outpatient prescription database (PharmaNet) [24] and vital statistics for cause of death (see Additional file 1: Table S1 for additional information on the data holdings). Individual consent was not required for the records, however the patient records/information was anonymized and de-identified prior to analysis. Ethics approval for the study was obtained from the University of British Columbia’s Ethics Committee.

### Study population

Individuals were eligible for inclusion in the cohort if they were a resident of British Columbia between January 1, 1997 to December 31, 2012 with incident HZ. To improve coding accuracy, we excluded visits or admissions with coexistent codes for varicella (ICD-9 052 and ICD-10 B01). An incident zoster case was defined as an enrollee with a HZ ICD-9 (053) or ICD-10 (B02) code in the primary or secondary position (i.e., all other) without any evidence of HZ or PHN within 12 months prior to this incidence. All 25 diagnostic codes (primary as well as secondary codes) in the hospitalization data were used to identify cases. If the first position (or diagnosis) was the primary reason for admission, all other positions from 2nd to 25th, if any, were categorized as the secondary diagnostic codes. To identify incident cases, we only included the first outpatient visit or hospitalization during the study period for each individual. In order to further ensure we were obtaining incident zoster cases only, enrollees with only PHN-specific ICD-9 (053.12, 053.13) or ICD-10 (B02.22, B02.23) were excluded. We conducted a sensitivity analysis and used the methodology proposed by Zhang et al. to increase positive predictive value of a HZ diagnosis in an administrative data source by defining incident herpes zoster as the presence of an ICD-9 or −10 code for herpes zoster plus receipt of antivirals, acyclovir, valacyclovir, famciclovir, within 7 days before or after the diagnostic code for HZ [25].

PHN was identified as those individuals with a first episode of zoster with a further zoster diagnostic code after 90 days with a relevant prescription for analgesia, anticonvulsant, or antidepressant therapy on the same day as the recorded consultation [26]. The presence of codes for non-specific neuralgia or for neurological complications of zoster after 90 days was also consistent with PHN. Immunosuppression status was identified by the presence of two diagnostic ICD-9 or ICD-10 codes on different days as an outpatient or inpatient within one year prior to the initial HZ diagnosis date. The following patients were considered as immunosuppressed: hematopoietic stem cell or solid organ transplantation; hematological malignancies such as Hodgkin’s lymphoma, multiple myeloma, acute leukemia, non-Hodgkin’s lymphoma; other hematological diseases, such as aplastic anemia, agranulocytosis, myelodysplastic syndrome; AIDS, advanced HIV infection; cancer, and other disorders involving immunodeficiency (see Additional file 1: Table S2 for ICD-9 and ICD-10 codes).

### Statistical analysis

For our primary analysis, we calculated incidence as the number of incident cases divided by the BC population. The overall crude annual incidence rates (number of events per 1000 population) of HZ and PHN was calculated by year and age group. We standardized the incidence by age and sex using the 2006 Canadian census data.

Varicella vaccines were introduced in Canada in 1998 but were implemented as part of routine immunization programs in British Columbia in September 2004 and January 2005 for susceptible kindergarten (age 6)/grade 6 students (age 12) and infants greater than or equal to 12 months of age, respectively [27]. At that time it was a one-dose program, but was later changed to a two-dose program January 1, 2012 [28]. The HZ vaccine, a live attenuated vaccine containing the Oka/Merck strain of varicella-zoster virus, was marketed in 2008 in Canada [29, 30]. We used a negative binomial regression model to assess the impact of varicella vaccination on zoster incidence during the three periods of the varicella vaccination program: the pre-licensure period (1997–1998), the period when the vaccine was available privately (1999–2004) and the publicly funded one-dose vaccination period (2005–2012). The model included age group, sex, immunosuppression status, indicator variables for varicella vaccine implementation, and calendar year. Interaction effects were assessed and sensitivity analysis was also performed for those aged 65 years or older. To evaluate risk factors for HZ, we calculated adjusted rate ratios for age, sex, immunosuppression status and varicella vaccine availability period using the regression model.

Finally, we evaluated the number and costs associated with hospitalizations, General Practioner (GP) visits, and treatment of HZ and PHN. Treatment for HZ was obtained by linking the prescription database to the hospitalization or GP office visit on that day and looking for antivirals, analgesics, antidepressants, corticosteroids within 90 days post-initial HZ visit. Each hospitalization inpatient record was assigned at least one methodology-specific resource intensity weight (RIW). The cost associated with acute care hospitalization was estimated by multiplying the highest RIW to the provincial estimates of the cost per weighted-case (CPWC) (i.e., hospitalization cost = RIW*CPWC). The cost associated with a GP visit was obtained from the amount paid in the MSP billing system. Treatment cost was defined as the total of drug cost submitted and the pharmacy professional fee submitted (i.e., PharmaNet cost = submitted drug cost + submitted professional fee). All costs were inflated by multiplying the ratio of the British Columbia consumer price index (CPI) for health care with the base year of 2013.

All statistical tests were two-tailed and *p =* 0.05 used to determine statistical significance. All analyses were undertaken using SAS 9.4 (SAS Institute Inc., Cary, NC) [31]. Bonferroni correction was applied in multiple comparisons and confidence intervals constructions, wherever applicable.

## Results

### Herpes zoster cases

From 1997 to 2012, there were 238,295 incident cases of herpes zoster in our study population, after excluding 201 cases with either co-existing varicella codes (*n =* 68) or only PHN specific codes (*n =* 133) (Table 1). The mean age of the zoster cases increased from 49.6 years in 1997 to 53.2 years in 2012 and there were more females than males (*n =* 138,855; 58.3 %). Among the herpes zoster cases, 9526 (4.0 %) were immunosuppressed at the time of diagnosis; only 0.2 % (*n =* 516) were vaccinated in our cohort.

**Table 1:** Profile of Herpes Zoster Cases by Varicella Vaccine Availability, BC 1997–2012

	Pre-licensure	Privately Funded	Publicly Funded	Overall
	1997–1998	1999–2004	2005–2012	1997–2012
Age				
0–9	1577 (6.7 %)	4562 (5.9 %)	3619 (2.6 %)	9758 (4.1 %)
10–19	1422 (6 %)	4649 (6.1 %)	6995 (5.1 %)	13066 (5.5 %)
20–29	2073 (8.8 %)	6314 (8.2 %)	11706 (8.5 %)	20093 (8.4 %)
30–39	2952 (12.5 %)	8743 (11.4 %)	13304 (9.7 %)	24999 (10.5 %)
40–49	3098 (13.1 %)	10550 (13.7 %)	17872 (13 %)	31520 (13.2 %)
50–59	3332 (14.1 %)	12544 (16.3 %)	25707 (18.7 %)	41583 (17.5 %)
60–69	3397 (14.4 %)	11306 (14.7 %)	25270 (18.3 %)	39973 (16.8 %)
70–79	3682 (15.6 %)	11049 (14.4 %)	19552 (14.2 %)	34283 (14.4 %)
80+	2089 (8.8 %)	7149 (9.3 %)	13738 (10 %)	22976 (9.6 %)
Unknown	12 (0.1 %)	24 (0 %)	8 (0 %)	44 (0 %)
Mean (IQR)	49.6 (33–69)	50.2 (34–69)	53.2 (39–69)	51.9 (36–69)
Gender				
Male	10141 (42.9 %)	32247 (41.9 %)	56917 (41.3 %)	99305 (41.7 %)
Female	13453 (56.9 %)	44579 (58 %)	80823 (58.7 %)	138855 (58.3 %)
Unknown	40 (0.2 %)	64 (0.1 %)	31 (0 %)	135 (0.1 %)
Health Authority				
Interior	4040 (17.1 %)	13659 (17.8 %)	24215 (17.6 %)	41914 (17.6 %)
Fraser	7470 (31.6 %)	24894 (32.4 %)	43316 (31.4 %)	75680 (31.8 %)
Vancouver Coastal	5684 (24.1 %)	17632 (22.9 %)	33518 (24.3 %)	56834 (23.9 %)
Island	4264 (18 %)	14231 (18.5 %)	26046 (18.9 %)	44541 (18.7 %)
Northern	1174 (5.0 %)	3667 (4.8 %)	7902 (5.7 %)	12743 (5.4 %)
Unknown	1002 (4.2 %)	2807 (3.7 %)	2774 (2 %)	6583 (2.8 %)
Immunosuppression Status (Any)				
Yes	905 (3.8 %)	3121 (4.1 %)	5500 (4.0 %)	9526 (4.0 %)
No	22729 (96.2 %)	73769 (95.9 %)	132271 (96.0 %)	228769 (96.0 %)
Zoster Vaccinated				
Yes	NA	NA	516 (0.4 %)	516 (0.2 %)
No	23634 (100 %)	76890 (100 %)	137255 (99.6 %)	237779 (99.8 %)

### Herpes zoster incidence

**Fig. 1:**
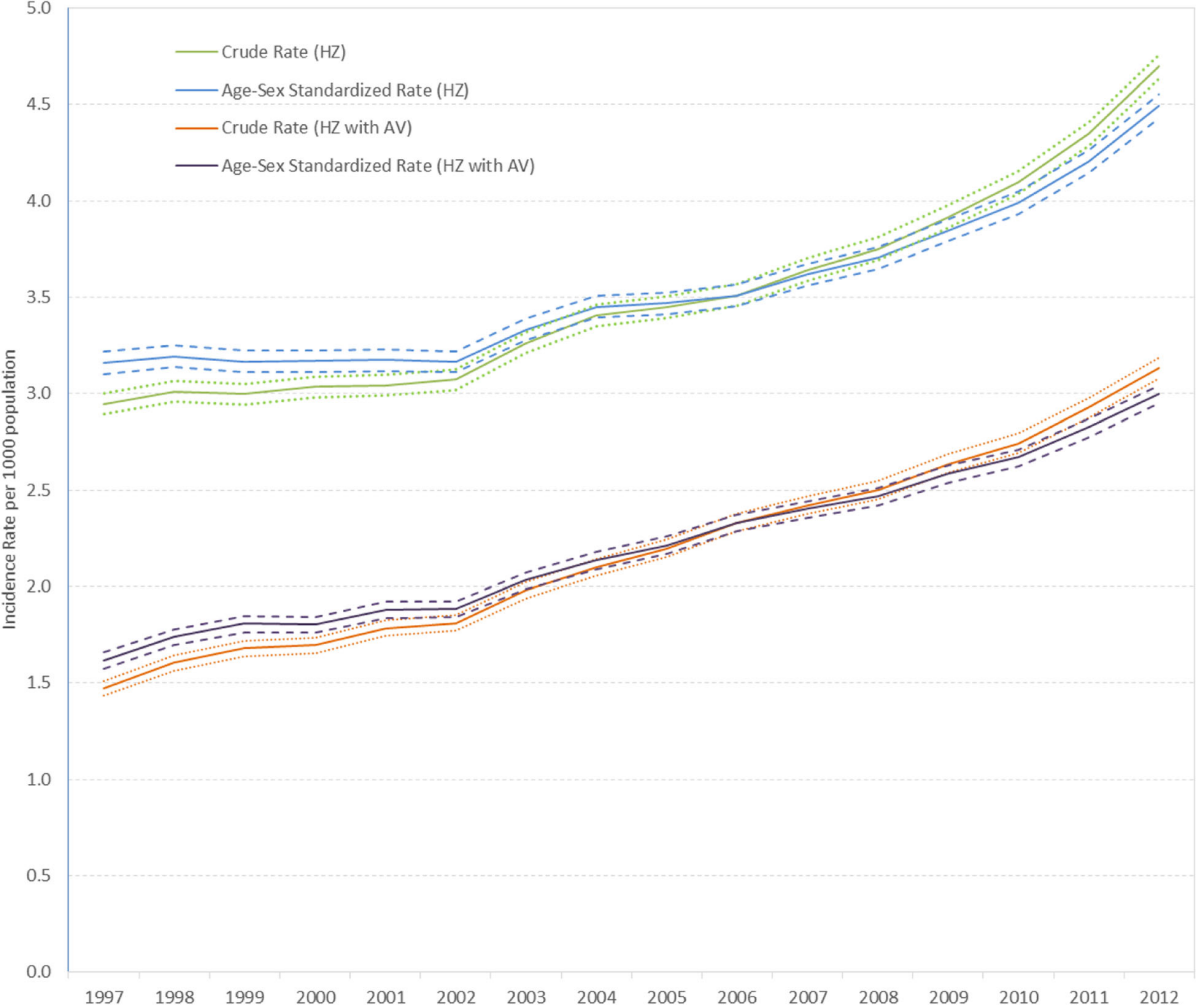
Crude and Age-Sex Standardized Herpes Zoster Incidence Rate and 95 % Confidence Interval by Year. The crude and age-sex adjusted rate of herpes zoster and 95 % confidence interval between 1997 and 2012 using two different definitions of incident zoster: as defined by ICD9/10 code and as the presence of an ICD-9/10 code for herpes zoster plus receipt of antivirals within 7 days before or after the diagnostic code for HZ

Crude HZ incidence increased with time from 2.9 per 1000 population in 1997 to 4.7 per 1000 population in 2012. As shown in Fig. 1, the age-sex standardized HZ rates also illustrated the increasing trend between 1997 (3.2 per 1000 population) and 2012 (4.5 per 1000 population) (see Additional file 1: Table S3).

**Fig. 2:**
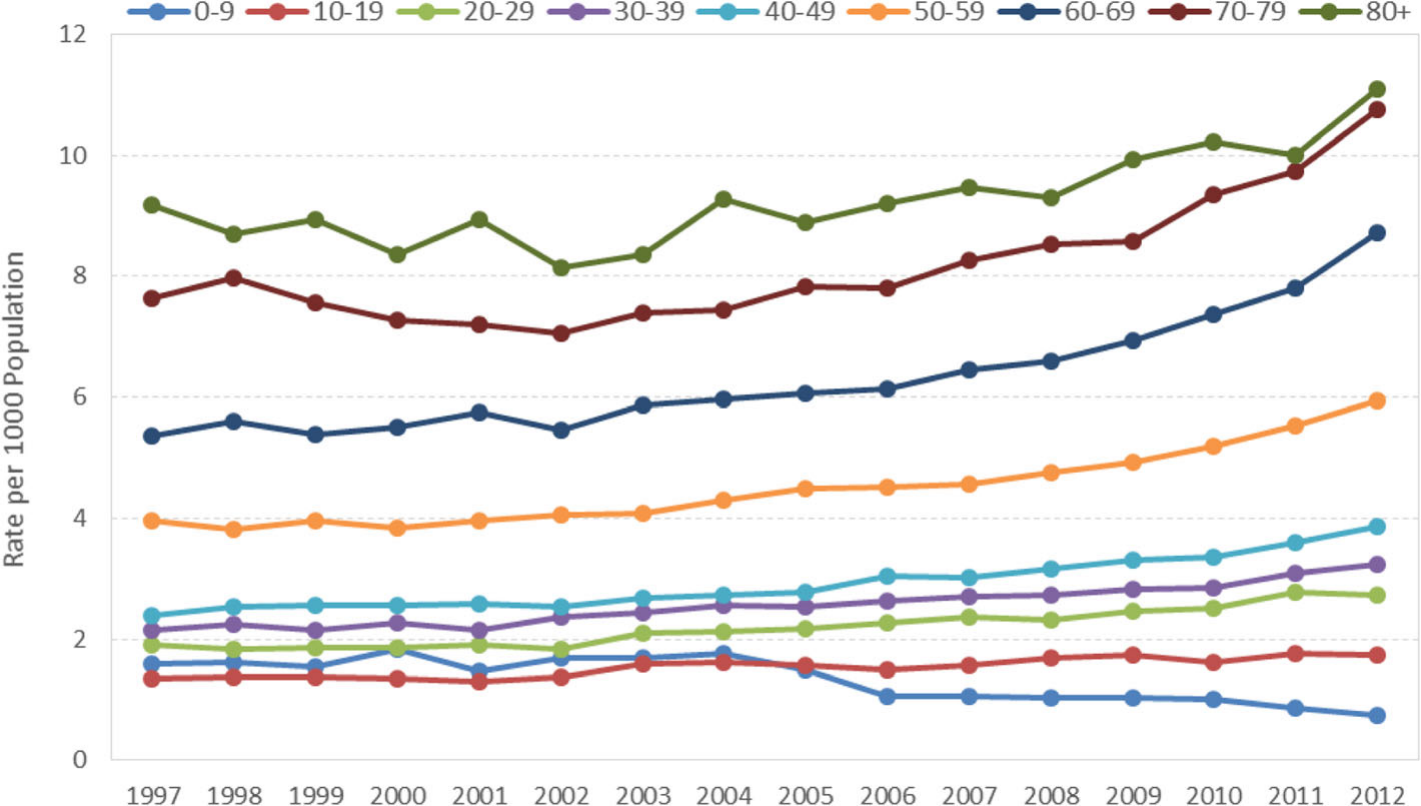
Herpes Zoster Incidence Rate, by Age Group and Year. The rate of herpes zoster between 1997 and 2012, by various age groups

HZ incidence rates were strongly age-related (Fig. 2). In 2012, the incidence in children and adolescents (<20 years) was 1.3/1000 population (95 % CI: 1.2–1.4), while in adults aged ≥50 years the incidence was 8.2/1000 population (95 % CI: 8.1–8.4). This trend was maintained each year during the whole study period. Over time, zoster incidence increased in all age groups, except 0–9 year age group which indicated a 50 % decrease since 2004 from 1.6 to 0.8 per 1000 population (Fig. 2). The highest increases in incidence from 1997 to 2012 were seen in age groups 40–49 years (2.4 to 3.9 per 1000 population, 63 % increase) and 60–69 years (5.4 to 8.7 per 1000 population, a 61 % increase). Although age group 80 years and up had the highest incidence rate, this age group experienced the lowest increase of 21 % from 9.2 to 11.1 per 1000 population.

The incidence was significantly higher among females compared to males across all the years (females: 3.35; 95%CI: 3.27, 3.43 vs males: 2.53; 95%CI: 2.46, 2.60 in 1997 and females: 5.48; 95%CI: 5.39, 5.58 vs males: 3.90; 95%CI: 3.82, 3.98 in 2012). This significant higher rate of zoster infection in females persisted in almost all age groups. There was a 19.3 % and 21.8 % increase in HZ during the publicly funded varicella vaccine period (3.869 per 1000 population) as compared to the privately funded period (3.244 per 1000 population) and the pre-licensure period (3.175 per 1000 population), respectively (Table 2).

**Table 2:** Age-Sex Standardized Mean Annual Incidence Rate by Varicella Vaccine Availability Periods

	Age-Sex Standardized Incidence Rate per 1000 Population (95 % Confidence Interval)			
	Pre-licensure (1997–1998)	Privately Funded (1999–2004)	Publicly Funded (2005–2012)	Overall (1997–2012)
HZ	3.175 (3.134, 3.217)	3.244 (3.221, 3.267)	3.869 (3.849, 3.890)	3.575 (3.561, 3.589)
HZ with Antiviral	1.678 (1.648, 1.709)	1.926 (1.908, 1.944)	2.571 (2.554, 2.587)	2.242 (2.231, 2.254)
PHN within 90 days	0.127 (0.119, 0.135)	0.169 (0.164, 0.175)	0.271 (0.266, 0.277)	0.221 (0.217, 0.224)
PHN within 30 days	0.104 (0.097, 0.112)	0.141 (0.136, 0.146)	0.232 (0.227, 0.237)	0.187 (0.184, 0.191)

In order to increase specificity for the HZ case definition, we conducted a sensitivity analysis of the case definition, which was HZ accompanied with antiviral taken within 7 days of diagnosis; the analysis of HZ incidence showed a similar pattern to the primary analysis (Fig. 1 and Additional file 1: Table S3).

### PHN incidence

**Fig. 3:**
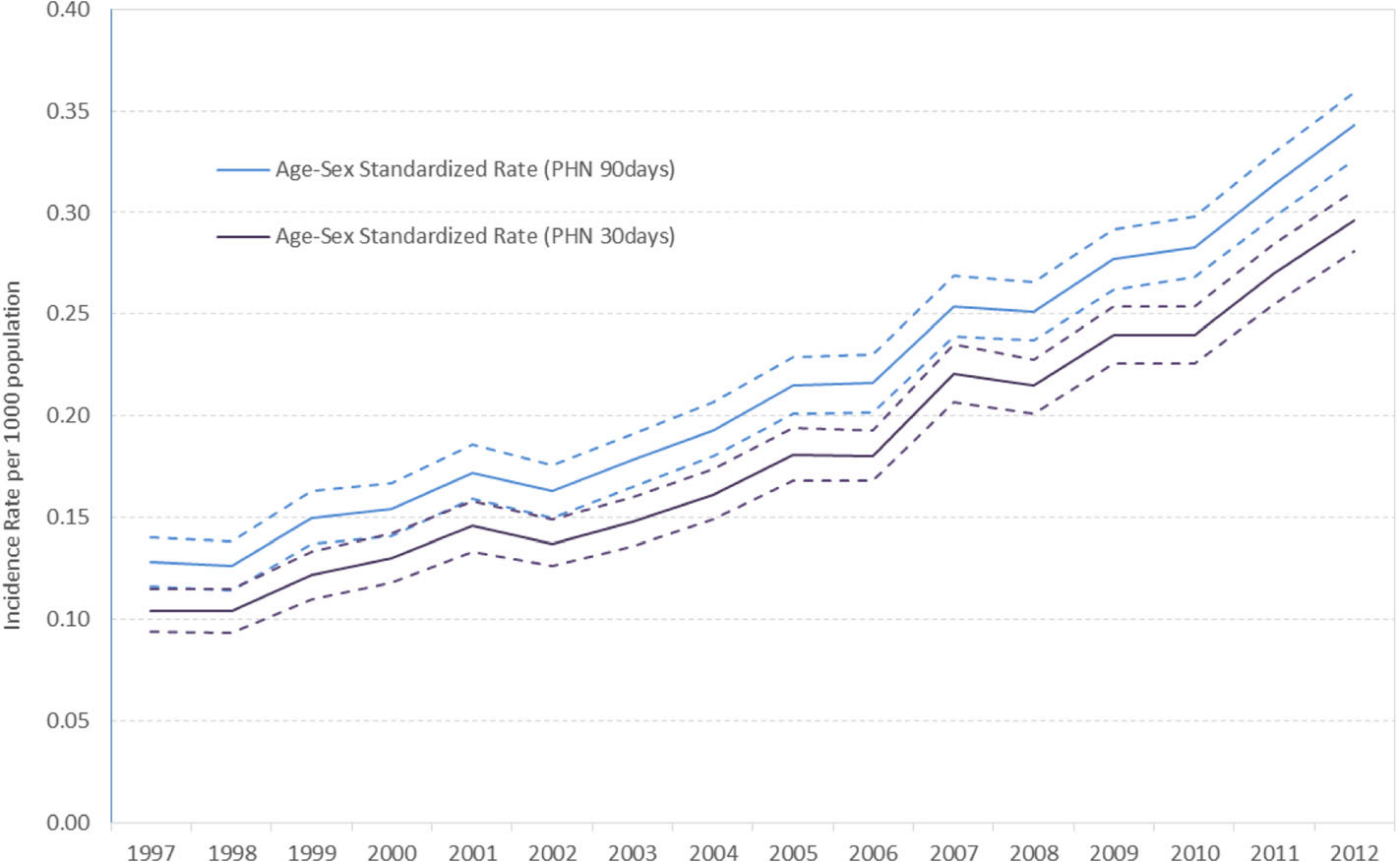
Age-Sex Standardized PHN Incidence Rate and 95 % Confidence Interval by Year. The age-sex adjusted rate of post-herpectic neuralgia between 1997 and 2012 using two different definitions of PHN: PHN diagnosed within 90 days post HZ diagnosis and within 30 days post HZ diagnosis

**Fig. 4:**
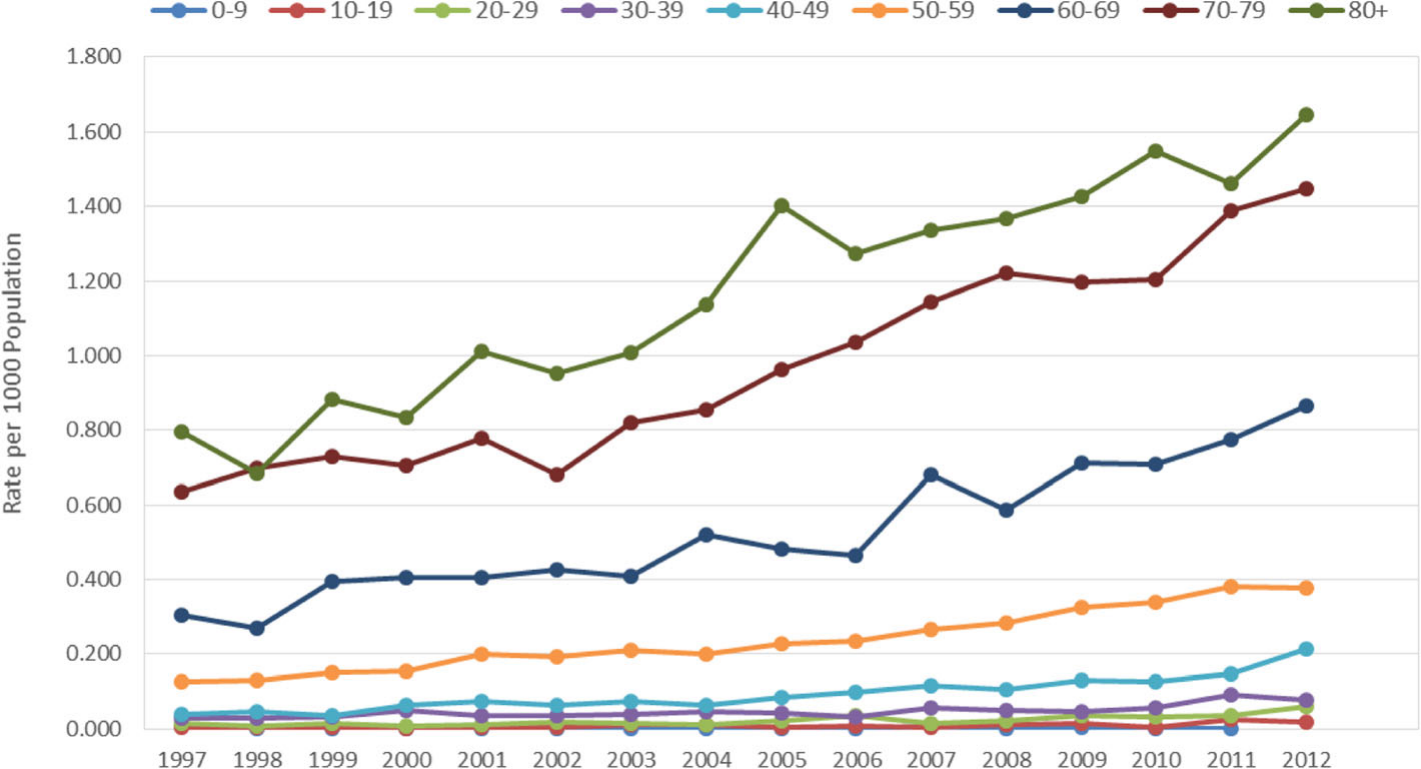
Post-herpectic Neuralgia Incidence Rate, by Age Group and Year. The rate of post-herpectic neuralgia between 1997 and 2012, by various age groups

Crude and age-sex standardized incidence rate of PHN demonstrated very similar patterns to the HZ incidence (see Additional file 1: Table S2 for the crude analysis). Figure 3 shows that the standardized rates increased almost 3-fold from 0.128 to 0.343 per 1000 population for PHN defined within 90 days post HZ diagnosis. We saw a similar result for our sensitivity analysis where PHN was defined within 30 days post HZ diagnosis (age adjusted rates increased from 0.104 to 0.296 per 1000 population (Fig. 3). All age groups showed increasing incidence over the years (Fig. 4). The highest increase in PHN incidence within 90 days post initial HZ from 1997 to 2012 was found in age groups 40–49 years (5.6-fold from 0.04 to 0.2 per 1000 population) and 10–19 years (4.5-fold from 0.004 to 0.02 per 1000 population). Although the age group of 80 years and up had the highest incidence rate, this age group experienced the lowest increase (2.1-fold from 0.8 to 1.6 per 1000 population). The incidence was significantly higher among females compared to males across all the years (females: 0.14; 95%CI: 0.12, 0.15 vs males: 0.08; 95%CI: 0.07, 0.10 in 1997 and females: 0.42; 95%CI: 0.40, 0.45 vs males: 0.32; 95%CI: 0.30, 0.34 in 2012). Although females had higher rates than males across all age groups, their differences were not significant across the years. Similar patterns were shown in our sensitivity analysis defined as PHN within 30 days post HZ diagnosis.

### Rate ratios comparisons on the impact of varicella vaccine availability on HZ incidence

**Fig. 5:**
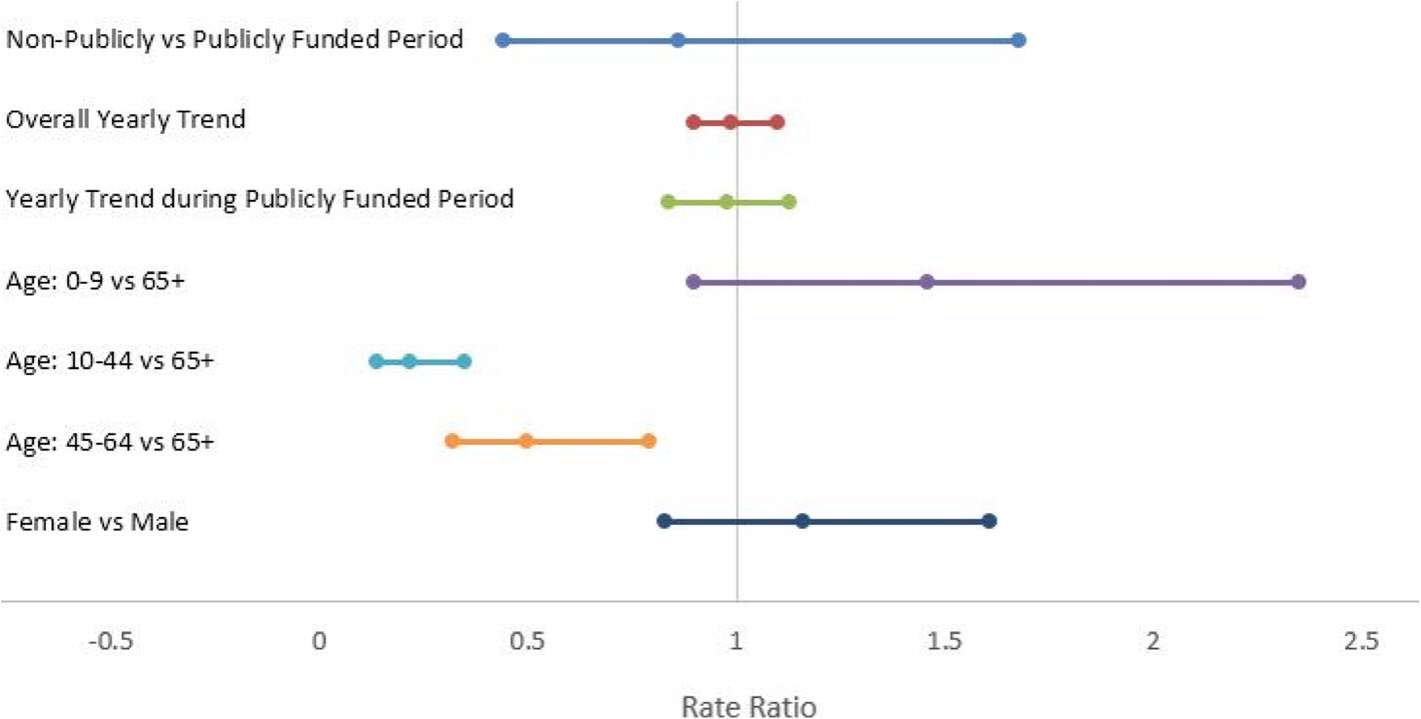
Rate Ratio on Herpes Zoster Incidence and 95 % Confidence Interval Using a Regression Model. The reference groups for the analyses included publicly funded period, age 65+ years and male gender

In our univariate analysis, the standardized mean annual HZ incidence rates were significantly higher during the publicly funded varicella vaccine period as compared to privately funded period (rate ratio 1.193; 95 % CI: 1.180, 1.205) and pre-licensure period (rate ratio 1.218; 95 % CI: 1.199, 1.238) (Additional file 1: Table S4). However, in our multivariate analysis, the risk of HZ during the publicly funded varicella vaccine period was not statistically different from the non-publicly funded period after controlling for the effects of age, gender and immunosuppressive status (Fig. 5, Additional file 1: Table S5). No significant interaction effects were found. Age remained as having a significant effect on the rate of HZ - the risk of HZ for those 65 years of age and over was 2 times (1/0.5) higher than for those in age group 45–64 years, and 4.5 times (1/0.22) higher than for those in age group 10–44 years. Although females show a 16 % higher risk of HZ than males, the effect was not statistical significant. We conducted a similar analysis restricted to those aged 65 years and over and the results were similar (data not shown).

### Hospital admissions, GP visits and treatment related to HZ and PHN

Table 3 shows that between 1997 and 2012, on a yearly basis, 2 % of the initial cases were diagnosed through hospitalization records, while the majority of HZ episodes were non-hospitalized. The trend over time is a slight decrease in the number of hospitalizations for treatment of HZ, and a slight increase in the number of GP visits per year with time. For those who were hospitalized the average age was 73.0 years (SD: 18.3), with a median age of 78 years. Majority of the patients were women (60.6 %), 2.7 % were pediatric (i.e., 0–19 years), 14.3 % were adults (20–59 years) and 82.9 % were 60 years and older. In contrast, the patients who visited their GPs, were younger - the average age was 60.2 years (SD: 19.2), with a median age of 63 years. Fifty eight percent were women, 3.9 % were pediatric (0–19 years), 38.7 % were adults (20–59 years) and 57.4 % were 60 years and older.

**Table 3:** Hospitalizations, General Practitioner Visits, and Treatment Costs^a^ Associated with HZ and PHN

	Pre-licensure	Privately Funded	Publicly Funded	Overall
	1997–1999	1999–2004	2005–2012	1997–2012
	(3 years)	(6 years)	(8 years)	(16 years)
Initial HZ Cases Identified Based on Hospitalization or GP Visits				
Hospitalization (Annual count, %)	409 (3.5 %)	296 (2.3 %)	276 (1.6 %)	300 (2 %)
Annual Cost	$4,747,242	$4,290,917	$5,428,559	$4,916,779
Cost/hospitalization Case	$11,607	$14,505	$19,678	$16,389
GP Visit (Annual count, %)	11408 (96.5 %)	12519 (97.7 %)	16946 (98.4 %)	14593 (98 %)
Annual Cost	$451,523	$475,046	$605,407	$537,286
Cost/GP Case	$40	$38	$36	$37
Number of Cases Treated (Annual count, %)^b^	4889 (41.4 %)	6281 (49 %)	9987 (58 %)	7960 (53.4 %)
Annual Treatment Costs	$750,370	$896,161	$1,091,457	$975,585
Cost/Rx Case	$153	$143	$109	$123
Overall Annual Cost	$5,949,134	$5,662,124	$7,125,422	$6,429,649
Overall Cost/Case	$503	$442	$414	$432
HZ Related Hospitalization or GP Visits within 90 days Post Initial HZ Diagnosis Hospitalization				
Annual Cases^c^ with ≥1 admission	109 (0.9 %)	84 (0.7 %)	93 (0.5 %)	91 (0.6 %)
Annual Cases with 0 admission	11708 (99.1 %)	12732 (99.3 %)	17129 (99.5 %)	14802 (99.4 %)
Annual Cost	$1,164,402	$963,319	$1,381,308	$1,197,449
Cost/Admission	$9,785	$10,763	$13,779	$12,149
GP Visits				
Annual Cases^c^ with ≥1 Visit	3207 (27.1 %)	3316 (25.9 %)	4588 (26.6 %)	3938 (26.4 %)
Annual Cases with 0 Visit	8610 (72.9 %)	9500 (74.1 %)	12634 (73.4 %)	10956 (73.6 %)
Annual Visits	8168	7602	12659	10201
Annual Visits with Rx^d^	1289	1458	2925	2170
Annual Visit Cost	$219,145	$218,276	$289,042	$253,768
Annual Rx Cost	$112,653	$122,106	$219,036	$169,389
Annual Cost (Visit + Rx)	$331,798	$340,382	$508,078	$423,157
Cost/Visit (including Rx cost)	$41	$45	$40	$41
PHN Related Hospitalization or GP Visits within 90 days Post Initial HZ Diagnosis Hospitalization				
Annual Cases^c^ with ≥1 admission	39 (0.3 %)	35 (0.3 %)	37 (0.2 %)	37 (0.2 %)
Annual Cases with 0 admission	11778 (99.7 %)	12780 (99.7 %)	17184 (99.8 %)	14857 (99.8 %)
Annual Admission	43	38	40	39
Annual Cost	$360,725	$539,429	$610,727	$552,740
Cost/Admission	$8,488	$14,196	$15,364	$14,016
GP Visits				
Annual Cases^c^ with ≥1 Visit	416 (3.2 %)	614 (4.8 %)	1192 (6.9 %)	878 (5.9 %)
Annual Cases with 0 Visit	11410 (96.6 %)	12201 (95.2 %)	16029 (93.1 %)	14015 (94.1 %)
Annual Visits	606	901	2015	1421
Annual Visits with Rx^e^	478	751	1514	1098
Annual Visit Cost	$5,693	$6,596	$19,048	$12,709
Annual Rx Cost	$17,011	$39,874	$91,544	$62,851
Annual Cost (Visit + Rx)	$22,704	$46,470	$110,592	$75,560
Cost/Visit	$37	$52	$55	$53

^a^All Costs were adjusted to 2013 British Columbia Consumer Price Index, Health and Personal Care

^b^Prescriptions on the same day of GP visits related to initial HZ treatment were antivirals (94.5 %), anticonvulsants (5.5 %), antidepressants (3.9 %), corticosteroids (2.6 %), NSAIDS (2.5 %), immunosuppressants (0.1 %), non-NSAID analgesia (0.0 %)

^c^Case refers to the number of patients with at least one related PHN or HZ hospital admission or GP visits 90 days after initial HZ diagnosis

^d^Prescriptions on the same day of GP visits related to HZ treatment were anticonvulsants (40.3 %), antivirals (38.6 %), antidepressants (18.3 %), NSAIDS (6.8 %), corticosteroids (6.5 %), immunosuppressants (0.2 %), non-NSAID analgesia (0.1 %)

^e^Prescription on the same day of GP visits related to PHN were anticonvulsants (61.7 %), antidepressants (27.9 %), NSAIDS (9.8 %), corticosteroids (8.7 %), antivirals (7.5 %), non-NSAID analgesia (0.2 %), immunosuppressant (0.2 %)

The annual cost of hospitalizing patients with HZ is approximately $4.9 million, compared to $537,286 for GP visits, resulting in an average cost per case of $16,389 for hospitalization and $37 for GP-related visit. Treatment was initiated in 53.4 % of the cases seen by GPs and the majority of the cases (95 %) received antivirals, thus when including costs associated with treatment, the annual cost per case of a GP-related visit would increase by an additional $123 per case for initial treatment of zoster infection. Subsequent visits to the GP or hospitalization within 90 days of the initial HZ diagnosis were very few. From 1997 to 2012, only 0.6 % of cases were re-admitted into the hospital, with an annual readmission cost of $12,149 per admission. In contrast, on average, 26.4 % of cases re-visited their GP within 90 days of their initial HZ diagnosis, costing an additional $253,768 per year to the healthcare system.

Between 1997 to 2012, there were a total of 588 (0.2 %) cases with at least one hospitalization related to PHN whereas the majority of the cases (total *N =* 14,054; (5.9 %)) were seen by GPs. On a yearly average, there were 39 hospitalizations and 1421 GP visits related to PHN. The number of hospital admissions per year over time has remained steady, but the annual number of GP visits for treatment of PHN has increased with time. For those who were hospitalized, the average age was 76.5 years (SD: 13.5), with a median age of 80 years and 62.9 % of the patients were women, 0.3 % were pediatric (0–19 years), 9.7 % were adults (20–59 years) and 90.0 % were 60 years and older. For patients who visited their GPs, the average age was 66.7 years (SD: 14.8), with a median age of 69 years and 60.4 % of the patients were women, 0.6 % were pediatric (0–19 years), 27.5 % were adults (20–59 years) and 71.9 % were 60 years and older.

Between 1997 and 2012, the total annual cost for PHN-related hospitalization and GP visits were over $552,740 and $12,709, respectively. Total costs per year for PHN that is treated in by a GP have risen significantly, from $5,693 to $19,048 per year. Prescription costs associated with outpatient treatment of PHN were estimated at $1.0 million (overall average $62,800/year) and again have risen considerably over time, from $17,011 to $91,544 per year. In contrast to HZ treatment, medications used for PHN were primarily anticonvulsants (61.7 %) and antidepressants (27.9 %).

## Discussion

In our study, over the 16 year period, we saw a 1.5-fold increase in HZ incidence regardless of the definitions used to identify herpes zoster. For young children between the ages of 0 and 9 years, the HZ incidence decreased abruptly from 1.8 to 0.8 per 1000 population in 2004, and has been steadily declining, likely reflecting the start of the publicly-funded varicella vaccination program which targeted children in kindergarten (age 6 years). Interestingly, despite the introduction of the varicella vaccination program in 2004 for 12 year olds, the age-specific HZ incidence held steady but did not decline for those within the 10–19 year old category. This is probably related to the fact that most of them will have had varicella infection already and have good immunity; for now too little time has elapsed to show a decrease in this age groups of HZ rates. Alternatively, the lack of effect on HZ rates may be related to a lower vaccine uptake rate in this age group than the kindergarteners or the fact that the vaccine effectiveness was reduced with the one dose program. On the positive side, zoster rates have not increased either, as they have for all other age categories after the age of 19 years. Although we saw a slight increase in zoster incidence in the 20–39 year old age groups, the largest increases were seen in the 40–49 (63 %) and the 60–69 year olds (61 %). The 80 year old group had the highest incidence in 2012 (11/1000 population), although their increase was only 21 % between 1997 and 2012. Our finding that HZ incidence increases with age has been reported in other studies and is thought to be related to immunosenescence, that is, decreasing ability to respond to the reactivated virus as the body ages [4, 19]. Pinchinat and colleagues [12] in their review of studies looking at the incidence of HZ across Europe showed that HZ incidence increases sharply with age, from around 1/1 000 children <10 years up to 10/1 000 people over 80 years of age. Similarly Kawai et al. found that age-specific incidence was similar across countries and rose sharply after the age of 50 years; their rates were similar to our study as they found rates of 6-8/1000 person-years at age 60 years and 8-12/1000 person-years at age 80 years [13].

Our observation of a higher zoster incidence in women has also been seen in other studies [7, 32–34]. Originally, investigators believed this difference was not a real phenomenon and it was likely due to women having greater health-seeking behavior. However, given that the difference between females and males occurs in all age groups, including children, and has been seen consistently in many studies using different methodologies, it is unlikely that this phenomenon is due to differential health-seeking behaviours. The biological reason behind this effect is unclear; it may be related to symptomatic zoster being more common in women, or gender differences in immune responses to the varicella antigen and loss of immunity over time [7, 35, 36].

In our study, the standardized rates seen in 2012 for zoster were within the range reported in other North American and European studies [12, 13]. In their systematic review Kawai et al. included 130 studies conducted in 26 countries. They found the incidence of HZ ranged from 3 to 5 per 1000 person-years [13]. Although the methodology used in these studies varied from prospective surveillance to use of retrospective administrative data to determine incidence, most of the studies showed a temporal increase in the incidence of HZ and PHN in the past several decades. The authors also observed that those studies which evaluated all age groups (rather than restricting the analyses to those over 60 years of age) and were conducted in the 50s to early 90s, showed lower HZ rates (HZ incidence of North American studies ranged 1.31 to 2.40 per 1000) [37, 38] than studies conducted 2000s (HZ incidence of North American studies ranged 3.82 to 5.79 per 1000) [35, 36, 39–41].

In our secondary analysis we evaluated the impact of the introduction of the publicly funded varicella vaccination program. Between 2007 and 2012, the average coverage rates in the 2 year old, kindergarden (6 year old) and grade 6 (12 year old) children was 83 %, 74 %, and 63 %, respectively [42]. Despite these uptake rates, a vaccine effect was not seen after adjusting for potential confounders which would have an effect on HZ incidence, including age, sex, and immunosuppression status. Previous older studies using survey data and modeling had suggested that the introduction of a routine varicella vaccination program could potentially increase the incidence of zoster infections due to less boosting of immunity by the wild type virus [16, 18, 19, 43, 44]. However, North American studies conducted in the last 5 years, using large databases have shown that although age-specific HZ incidence is increasing, it started increasing before the introduction of widespread varicella vaccination. Hales et al. [45] conducted a retrospective study using Medicare claims to examine the link between herpes zoster incidence in the US population older than 65 years and childhood varicella vaccination. They found that age and sex standardized HZ incidence increased 39 % from 10.0 per 1000 person-years in 1992 to 13.9 per 1000 person-years in 2010. They used Poisson regression analysis to compare HZ trends during 3 periods of varicella vaccination program implementation: preimplementation (1992 to 1995), early implementation (1996 to 1999), and full implementation (2000 to 2010). The authors also found that the rise in HZ incidence predated 1996, when the U.S. Advisory Committee on Immunization Practices first recommended routine varicella vaccination for children aged 12 to 18 months, and HZ incidence did not accelerate after full implementation of the varicella vaccination program, when vaccine coverage reached 90 % and varicella incidence decreased by 90 %. The authors of this paper also looked at HZ incidence in US states that had low varicella vaccine coverage rates compared to high coverage rates; they found that state varicella vaccination coverage had no effect on HZ incidence concurrently or 10 years later (RR, 0.9998 [CI, 0.9993 to 1.0003]) after adjusting their model for sex, age, and calendar year [44]. Using medical claims data, Leung and colleagues [38] evaluated HZ incidence in all persons enrolled in the MarketScan® databases (Thomas Reuters, Ann Arbor, MI). They used similar definitions as our study and defined incident HZ as an enrollee of any age in the MarketScan® database with an outpatient claim bearing a HZ ICD-9 code (053.xx) in the primary or secondary diagnostic position. From 1993 through 2006, HZ incidence also increased 98 % from 1.7 (95%CI: 1.6-1.7) in 1993 to 4.4 (95 % CI: 4.3-4.4) in 2006. The increases occurred among all age strata and both sexes, although it increased more rapidly among females. Their results suggested greater increase in HZ rates between 1993 and 1996, prior to introduction of the varicella vaccination program. They also did not find any variation in HZ incidence by state varicella vaccination coverage rates. An interesting observation by the authors was that adults with dependents less than 12 years of age had lower HZ incidence at the outset of the varicella vaccination program compared with adults without dependents. However, the incidence in both groups became similar as the program progressed, suggesting that the introduction of the varicella vaccination program has not influenced HZ incidence in the general population, but it may have affected specific groups or households. In a recent Canadian epidemiologic study, Russell et al. [40] used multiple linked datasets to determine the incidence of HZ in Alberta, Canada and the impact of the varicella vaccination program, which has been publicly funded since 2002. Similar to our study, they showed that crude rates of medically attended HZ episodes increased over the interval of 1994–2010. Herpes zoster rate was 3.5 per 1000 person-years in 1994, 3.8/1000 person-years in 1998, 4.0/1000 person-years in 2001 and 4.5/1000 person-years by 2010. Rates were higher among females than males over the entire interval, and increased more rapidly for females than males. As in our study, prior to the publicly-funded varicella vaccination program in 2002, all age groups experienced increasing annual rates of HZ, but a sharp decline was seen in those less than 10 years of age for 2002–2010, the period in which varicella vaccination was publicly funded by the government. Other recent studies conducted in Australia [46], Japan [47] and Taiwan [48, 49] also have found that zoster rates were increasing before the introduction of their varicella vaccination programs. Although our study findings is in agreement with these studies, our data should be interpreted with caution. The varicella impact model we used may not capture the influence on zoster of varicella vaccination, because time periods we used were based on time of varicella vaccination policy changes and we did not allow for the impact of that vaccination on chickenpox rates to be seen. Because we did not allow for a lag time to see reductions in childhood varicella rates after introduction of a new vaccination program, we may have missed the true impact of childhood varicella vaccination program on HZ rates.

In our study we saw a very low PHN incidence of 0.343 per 1000 persons compared to the SPS trial [29], which showed the PHN incidence to be 1.38 per 1000 in the placebo group. In the systematic review conducted by Kawai et al., the risk of developing PHN ranged widely from 5 % to 30 % because of the different definitions used by researchers to classify duration, severity of pain and also because of differing comorbid illnesses, age and other underlying risk factors from one study to another [13]. The low incidence of PHN seen in our study is comparable to other studies using administrative databases to estimate PHN incidence; these studies are more likely to report a lower estimated risk of PHN compared with prospective studies (2.6 % to 6.9 %) [50–53]. This is likely the result of misclassification bias when using billing codes rather than actual medical records to delineate PHN. Further, the definition of PHN varies widely from one study to another and therefore, the incidence varies depending on whether the study uses the 90 day or 30 definition, making the direct comparison difficult across studies. Regardless of the definition, we did see a significant increase in the incidence of PHN between 1997 and 2012. Given that the definition of PHN included a diagnosis of zoster, we had expected to see a rise in PHN incidence but the magnitude of a 3-fold increase was a surprise. Although some of this increase is likely real and the result of the increase in HZ rates, we feel it may also be related to increasing recognition and awareness by the public and medical community about PHN being a common complication of zoster. Like previous studies, we also showed that the highest incidence of PHN was in the older individuals, especially those over the ages of 70 years, but in terms of increasing incidence with time, we saw the largest increase in those 40–49 years and 10–19 years. Understanding of the risk factors for PHN has evolved over the years; not only are older age, greater pain and rash severity, and presence of a prodrome known to be risk factors for PHN, but newer evidence would suggest immunosuppression, diabetes and trauma to also be risk factors for PHN [4, 54]. It may be that the younger cohort are more immunosuppressed due to specific diseases or medications, or the proportion of young adults with diabetes is increasing, or perhaps they or their parents are more prone to seeking medical attention for PHN symptoms than the elderly.

Between 1997 and 2012, only 0.6 % (6 per 1000 population) of our HZ cases were hospitalized, and over time we saw a slight decrease in the hospitalization rate from 0.9 % in 1997 to 0.5 % in 2012. Studies that have reported on hospitalization rates for HZ show a wide variation with reported rates ranging from 2 to 25/100 000 person-years in studies examining all ages [13]; the variation is probably related to differing admission criteria and whether studies used the HZ primary code or all codes for diagnosis. In our study, we saw that the mean age of patients hospitalized was higher and in the mid-seventies compared to those being seen by their GPs. Although we did not breakdown our data by age, studies have shown that hospitalization rates increase with age. For example, Jackson et al. reported hospitalization rates of 10 per 100,000 in adults 60–69 years of age but 65 per 100,000 in adults 80 years old or above [55]. Rates of hospitalization, in an Australian study, showed similar increases from 13 per 100,000 to 96 per 100,000 in adults 60–64 years of age and those ≥80 years of age respectively [56].

In our dataset, despite seeing a slight decrease in the number of hospitalizations for the treatment of HZ, we saw a significant increase in hospitalization costs over time. We believe, this may be related to more complicated cases being admitted for treatment while uncomplicated cases are being treated as outpatients, as we also saw an increase in the number of cases seen per year by GPs with HZ. Ninety-five percent of our HZ patients were treated with antivirals, which is similar to rates seen in studies conducted in Germany (71 %) [57], Italy (79 %) [51], and France (94 %) [58]. In general per case treatment costs of inpatients with HZ are approximately $16,000 and outpatients is around $40 per GP visit (an additional $123 if you include medications). With respect to PHN, we saw that most PHN cases were being treated on an outpatient basis, again with an increase over time with respect to the number of cases being seen by GPs. Like HZ, we saw an increase in the treatment costs associated with PHN. Costs associated for PHN treatment in the hospital is approximately $14,000 per case and for outpatients is $53 per visit. Given the numbers of patients who have a diagnosis of PHN, the annual cost of visiting a GP and receiving treatment is $75,560 to the healthcare system. It is difficult to compare cost data to other studies as the approaches used to calculate costs are so different; some studies look at direct and indirect costs while others, like ours look at direct costs only; some look at outpatient visits only, while other studies look at all medical expenses. Gauthier et al. estimated the mean total cost in their study of inpatient to be £103 ($206) per HZ case and £397 ($794) per HZ case for outpatient care [36]. This is in contrast to our study which showed higher hospitalization costs rather than outpatient costs. In an Italian study, the mean inpatient treatment costs for HZ was €2592 ($4147) while outpatient costs were €123 ($197) [51]. While the trends are similar to our study – higher costs for hospital visits – our costs were much higher for HZ hospitalization ($16,389) but similar for outpatient costs ($160).

Although our data with respect to the increase in HZ and PHN rates over time agree with previously reported studies, this may be an artifact given that ours is an epidemiological study using large datasets, and we were not able to directly access the accuracy of the administrative database claims through record reviews. As with all studies using administrative data, we used billing codes meant for physician billing as our basis to delineate zoster and PHN diagnosis. Because of this, changes in coding or coding errors could have led to an over- or under-estimation of the results. To try and overcome some of these issues and increase specificity, we used alternative definitions and found similar results to our main analysis. Our study was limited by the data available in the databases so if patients did not approach a medical facility for their zoster infection or PHN treatment, we would not have captured these events or their costs in our analyses, thereby underestimating their incidence; this may be particularly true around PHN-related costs as patients could have had alternative treatments for pain control. It is also possible that increasing HZ and PHN rates are simply due to improved coding and awareness. This may be especially true after the herpes zoster vaccine was marketed in 2008 as both the public and the medical community have been better educated on signs and symptoms of herpes zoster, the use of antivirals and analgesics for its treatment and complications arising from zoster infection, particularly PHN. Around the time the zoster vaccine was marketed in Canada, pharmacists received the authority to immunize and one of the vaccines they are heavily promoting to their clients is the herpes zoster vaccine, further enhancing awareness of the disease. However having said that, we know that between 2008 and 2012, the herpes zoster vaccine was marketed as a freezer-stable product and the uptake rate of the vaccine was less than 10 %. Although we adjusted for age and sex, we are not able to control for all the potential confounders which may have affected the HZ and PHN rates. In particular, the proportion of the population which has comorbidities such as diabetes mellitus (a known risk factor for HZ), and immunocompromising conditions such as solid organ transplants, cancer, leukemia/lymphoma, or being on medications that decrease T-cells (TNF-alpha inhibitors, DMARDS, corticosteroids) has increased over time, leading to increased numbers of patients being at risk for HZ/PHN [59]. Finally, as with all epidemiological studies, there may be additional risk factors for zoster and PHN that we are unaware of at the present time and these would not have been adjusted for in our study.

## Conclusions

In conclusion, we found the incidence of zoster and PHN is increasing with time. Although there are well known risk factors, such as age, female gender and being immunocompromised, which are contributing to the increased incidence seen with time, there are obviously risk factors present which are more difficult to tease out, such as trauma, psychological stress, race, and family history. Understanding the epidemiology and risk factors of HZ is critical for better targeting of treatment and prevention strategies.

## Additional files

Additional file 1:
**Table S1.** Description of the datasets used for data linkage. **Table S2.** Definitions for immunosuppression. **Table S3.** Crude and Age-Sex Standardized Incidence Rate and 95 % Confidence Interval by Year. **Table S4.** Rate Ratio of Age-Sex Standardized Mean Annual Incidence Rate and 95 % Confidence Interval with Bonferroni Correction for Multiple Comparison. **Table S5.** Rate Ratio on Herpes Zoster Incidence and 95 % Confidence Interval Using a Regression Model. (DOCX 28 kb)

## Abbreviations

HZ: Herpes zoster
HZO: Herpes zoster ophthalmicus
PHN: Post-herpetic neuralgia
TIA: Transient ischemic attack
VZV: Varicella zoster virus
