# Supplementary material for: Increasing incidence associated with herpes zoster infection in British Columbia, Canada
Source: BMC Infect Dis. 2016 Oct 20;16:589. doi: 10.1186/s12879-016-1898-z (PMC5073843; doi:10.1186/s12879-016-1898-z)
Supplement: Additional file 1: — Table S1. Description of the datasets used for data linkage. Table S2. Definitions for immunosuppression. Table S3. Crude and Age-Sex Standardized Incidence Rate and 95 % Confidence Interval by Year. Table S4. Rate Ratio of Age-Sex Standardized Mean Annual Incidence Rate and 95 % Confidence Interval with Bonferroni Correction for Multiple Comparison. Table S5. Rate Ratio on Herpes Zoster Incidence and 95 % Confidence Interval Using a Regression Model. (DOCX 28 kb) [file 12879_2016_1898_MOESM1_ESM.docx]

**Supplementary Material**

Table 1. Description of the datasets used for data linkage

| Data Set: Medical Services Plan (MSP) Payment Information File | Description: Data on medically necessary services provided by fee-for-service practitioners to individuals covered by the Medical Services Plan (MSP), BC's universal insurance program. Practitioners are separated into: physicians, supplementary benefit practitioners (physiotherapists, massage practitioners, naturopathic physicians, etc.), and out-of-province practitioners.  Date Range: April 1, 1985 onwards |
| --- | --- |
| Data Set: Discharge Abstract Database (Hospital Separations) | Description: Data on discharges, transfers and deaths of in-patients and day surgery patients from acute care hospitals in BC.  Date Range: April 1, 1985 onwards |
| Data Set: PharmaNet | Description: The PharmaNet system is an online, real-time system that captures all prescriptions for drugs and medical supplies dispensed from community pharmacies in BC as well as prescriptions dispensed from hospital outpatient pharmacies for patient use at home. In addition, physicians may record medications provided to patients during an office, clinic or emergency department visit. The recording of medications by physicians is not mandatory at this time; therefore this data is not complete. PharmaNet supports drug dispensing, drug monitoring and claims processing.  Inclusions: All prescription medications and supplies dispensed by community pharmacies in BC; Prescriptions dispensed from hospital outpatient pharmacies for patient use at home; Medications provided to patients during a physician office, clinic or emergency department visit (not mandatory at this time); Patient demographic data: Personal Health Number, name, address and date of birth, reported drug allergies; Drug information and drug interaction evaluations; PharmaCare and patient paid prescription claim information for drugs, dispensing fees, special services fees  Exclusions: Medications administered to patients when admitted to hospital; Drugs dispensed to hospital in-patients; Antiretroviral medications dispensed from the Centre of Excellence in HIV/Aids at St. Paul’s Hospital; Medications purchased without a prescription by the patient (e.g. Over the counter medications, herbal products, vitamins); Information regarding third party coverage; as a result it is not apparent if the patient paid amount was paid by the patient or a third party insurer; Third party paid amounts.  Date range: January 1, 1996 onwards |
| Data Set: Vital Statistics Deaths | Description: Information on all deaths registered in the province of BC.  Date range: January 1, 1985 onwards |
| Data Set: Consolidation File (MSP Registration & Premium Billing) | Description: The Consolidation File is Population Data BC's central demographics file for research requests. It contains basic demographics such as age and sex, geo-codes indicating location of residence, and registration data.  Date Range: January 1, 1986 onwards |

Table 2. Definitions for immunosuppression

| Condition Grouping | ICD9 | ICD10 | Description |
| --- | --- | --- | --- |
| Blood-related | 284.0-284.9 | D60,D61 | Aplastic anemia |
|  | 287.3-287.4 | D473,D693,D694,D695 | Primary and secondary thrombocytopenia |
|  | 288.0-288.9 | D70,D71,D72,D76 | Diseases of white blood cells |
|  | 289.4-289.59 | D731,D732,D5702,D57212,D57412,D7381,D733,D734,D75,D7389 | Diseases of spleen |
|  | 289.8-289.9 | D685,D686,D7581,D7582,D7589,D474,D892,D759 | Other specified and unspecified diseases of blood-forming organs |
| Cancer | 140-149 | C000,C001,C002,C003,C004,C005,C006,C008 | Malignant neoplasm of lip, oral cavity, pharynx |
|  | 150-159 | C153,C154,C155,C158,C159 | Malignant neoplasm of digestive organs & peritoneum |
|  | 160-165 | C300,C301,C310,C311,C312,C313,C318,C319 | Malignant neoplasm of respiratory & intrathoracic organs |
|  | 170-172 | C4000,C4010,C4020,C4030,C410,C411,C412,C413,C414,C419,C478,C490,C4910,C4920,C493,C494,C495,C496,C498,C430,C4310,C4320,C4330,C4331,C4339,C434,C4359,C4360,C4370,C438,C439,D030,D0310,D0311,D0312,D0320,D0321,D0322,D0330,D0339,D034,D0351,D0352,D0359,D0360,D0361,D0362,D0370,D0371,D0372,D038,D039 | Malignant neoplasm of bone, connective tissue, melanoma |
|  | 174-175 | C50019,C50119,C50219,C50319,C50419,C50519,C50619,C50819,C50919,C50029,C50929 | Malignant neoplasm of the breast |
|  | 179-189 | C55,C530,C531,C538,C539,C58,C540,C541,C542,C543,C548,C549,C569,C5700,C5710,C5720,C573,C574,C574,C510,C511,C512,C519,C52,C577,C578,C579,C61,C6200,C6210,C6290,C600,C601,C602,C608,C609,C6300,C6310,C632,C637,C638,C639,C670,C671,C672,C673,C674,C675,C676,C677,C678,C679,C649,C659,C669,C680,C681,C688,C689 | Malignant neoplasm of genitourinary organs |
|  | 190-199 | C6900,C6910,C6920,C6930,C6940,C6950,C6960,C6980,C6990,C710,C711,C712,C713,C714,C715,C716,C717,C718,C719,C700,C701,C709,C720,C721,C7250,C729,C729,C73,C7490,C750,C751,C752,C753,C754,C755,C758,C759,C760,C761,C762,C763,C7640,C7650,C768,C770,C771,C772,C773,C774,C775,C778,C779,C7800,C781,C782,C7839,C784,C785,C786,C787,C787,C7889,C7900,C7911,C7919,C792,C7931,C7932,C7949,C7951,C7952,C7960,C7970,C7981,C7982,C7989,C800,C801,C802 | Malignant neoplasm of other and unspecified sites |
|  | 200-208 | C831,C833,C835,C837,C838,C846,C847,C810,C811,C812,C813,C814,C817,C819,C829,C840,C841,C844,C858,C914,C960,C962,C96A,C964,C969,C96Z,C900,C901,C902,C903,C888,C910,C911,C91Z,C919,C920,C921,C922,C923,C924,C925,C929,C92Z,C930,C931,C939,C93Z,C940,C942,C943,C948,D45,C950,C951,C959, | Malignant neoplasm of lymphatic and hematopoietic tissue |
|  | 235-238 | D37,D38,D39,D40,D41,D43,D44,D45,D46,D47,D48,C944,Q850 | Neoplasms of uncertain behavior |
|  | 239 | D49 | Neoplasms of unspecified nature |
|  | 99.25 | 3E03305,3E04305 | Injection or infusion of cancer chemotherapeutic substance |
|  | V58.0 | Z510 | Radiotherapy |
|  | V58.11 | Z5111 | Encounter for antineoplastic chemotherapy |
| HIV | 042 | B20 | HIV infection and disease |
|  | 79.53 | B9735 | Human immunodeficiency virus, type 2 [HIV-2] |
| Transplant | 99.28 | 3E00X0M,3E0130M,3E0230M,3E03303,3E0330M,3E04303,3E0430M,3E05303,3E0530M,3E06303,3E0630M | Injection or infusion of biological response modifier [BRM] as an antineoplastic agent |
|  | V42.0-1; V42.3-9 | Z940,Z941,Z945,Z946,Z947,Z942,Z944,Z948,Z949 | Organ or tissue replaced by transplant (excludes heart valve) |

Table 3. Crude and Age-Sex Standardized Incidence Rate and 95% Confidence Interval by Year

|  | **Crude Incidence Rate per 1000 Population and 95% Confidence Interval** | | | |
| --- | --- | --- | --- | --- |
|  | **HZ** | **HZ with Antiviral^1^** | **PHN within 90days** | **PHN within 30days^2^** |
| **1997** | 2.947 ( 2.894, 3.001 ) | 1.471 ( 1.434, 1.509 ) | 0.111 ( 0.101, 0.122 ) | 0.091 ( 0.082, 0.101 ) |
| **1998** | 3.012 ( 2.959, 3.067 ) | 1.605 ( 1.566, 1.645 ) | 0.111 ( 0.102, 0.122 ) | 0.092 ( 0.083, 0.102 ) |
| **1999** | 2.997 ( 2.944, 3.051 ) | 1.679 ( 1.640, 1.720 ) | 0.134 ( 0.123, 0.146 ) | 0.109 ( 0.099, 0.119 ) |
| **2000** | 3.034 ( 2.981, 3.088 ) | 1.695 ( 1.656, 1.736 ) | 0.140 ( 0.129, 0.152 ) | 0.119 ( 0.108, 0.130 ) |
| **2001** | 3.044 ( 2.991, 3.098 ) | 1.784 ( 1.743, 1.825 ) | 0.159 ( 0.147, 0.172 ) | 0.134 ( 0.124, 0.146 ) |
| **2002** | 3.073 ( 3.020, 3.127 ) | 1.811 ( 1.771, 1.853 ) | 0.153 ( 0.142, 0.166 ) | 0.129 ( 0.118, 0.140 ) |
| **2003** | 3.262 ( 3.208, 3.318 ) | 1.979 ( 1.937, 2.023 ) | 0.170 ( 0.158, 0.184 ) | 0.142 ( 0.131, 0.154 ) |
| **2004** | 3.405 ( 3.349, 3.461 ) | 2.100 ( 2.056, 2.144 ) | 0.187 ( 0.175, 0.201 ) | 0.156 ( 0.145, 0.169 ) |
| **2005** | 3.447 ( 3.391, 3.503 ) | 2.196 ( 2.152, 2.242 ) | 0.212 ( 0.198, 0.226 ) | 0.179 ( 0.166, 0.192 ) |
| **2006** | 3.510 ( 3.455, 3.567 ) | 2.330 ( 2.284, 2.376 ) | 0.216 ( 0.202, 0.230 ) | 0.181 ( 0.168, 0.194 ) |
| **2007** | 3.644 ( 3.587, 3.701 ) | 2.422 ( 2.376, 2.469 ) | 0.258 ( 0.243, 0.273 ) | 0.224 ( 0.210, 0.239 ) |
| **2008** | 3.751 ( 3.694, 3.809 ) | 2.501 ( 2.454, 2.548 ) | 0.258 ( 0.243, 0.273 ) | 0.220 ( 0.206, 0.234 ) |
| **2009** | 3.917 ( 3.859, 3.976 ) | 2.637 ( 2.590, 2.686 ) | 0.287 ( 0.272, 0.304 ) | 0.249 ( 0.235, 0.264 ) |
| **2010** | 4.095 ( 4.036, 4.155 ) | 2.744 ( 2.696, 2.793 ) | 0.297 ( 0.282, 0.314 ) | 0.252 ( 0.238, 0.268 ) |
| **2011** | 4.349 ( 4.289, 4.410 ) | 2.927 ( 2.878, 2.978 ) | 0.335 ( 0.319, 0.353 ) | 0.288 ( 0.272, 0.304 ) |
| **2012** | 4.697 ( 4.634, 4.760 ) | 3.132 ( 3.081, 3.184 ) | 0.372 ( 0.354, 0.390 ) | 0.320 ( 0.304, 0.337 ) |
|  | **Age-Sex Standardized Incidence Rate per 1000 Population and 95% Confidence Interval** | | | |
|  | **HZ** | **HZ with Antiviral^1^** | **PHN within 90days** | **PHN within 30days^2^** |
| **1997** | 3.158 ( 3.099, 3.216 ) | 1.618 (1.576, 1.661 ) | 0.128 (0.116, 0.140) | 0.104 (0.094, 0.115) |
| **1998** | 3.194 ( 3.136, 3.252 ) | 1.738 (1.695, 1.781 ) | 0.126 (0.114, 0.138) | 0.104 (0.093, 0.115) |
| **1999** | 3.167 ( 3.110, 3.224 ) | 1.808 (1.764, 1.851 ) | 0.150 (0.137, 0.163) | 0.122 (0.110, 0.133) |
| **2000** | 3.168 ( 3.112, 3.225 ) | 1.802 (1.759, 1.845 ) | 0.154 (0.141, 0.167) | 0.130 (0.118, 0.142) |
| **2001** | 3.173 ( 3.116, 3.229 ) | 1.882 (1.839, 1.926 ) | 0.172 (0.159, 0.186) | 0.146 (0.133, 0.158) |
| **2002** | 3.164 ( 3.109, 3.220 ) | 1.883 (1.840, 1.926 ) | 0.163 (0.150, 0.176) | 0.137 (0.126, 0.149) |
| **2003** | 3.332 ( 3.275, 3.388 ) | 2.033 (1.989, 2.078 ) | 0.178 (0.165, 0.191) | 0.148 (0.136, 0.160) |
| **2004** | 3.450 ( 3.393, 3.507 ) | 2.135 (2.091, 2.180 ) | 0.193 (0.180, 0.207) | 0.161 (0.149, 0.174) |
| **2005** | 3.470 ( 3.413, 3.526 ) | 2.214 (2.169, 2.260 ) | 0.215 (0.201, 0.229) | 0.181 (0.168, 0.194) |
| **2006** | 3.509 ( 3.453, 3.566 ) | 2.329 (2.284, 2.375 ) | 0.216 (0.202, 0.230) | 0.180 (0.168, 0.193) |
| **2007** | 3.618 ( 3.562, 3.675 ) | 2.403 (2.357, 2.449 ) | 0.254 (0.239, 0.269) | 0.221 (0.207, 0.235) |
| **2008** | 3.704 ( 3.647, 3.761 ) | 2.466 (2.420, 2.513 ) | 0.251 (0.237, 0.266) | 0.215 (0.201, 0.228) |
| **2009** | 3.846 ( 3.789, 3.904 ) | 2.585 (2.538, 2.632 ) | 0.277 (0.262, 0.292) | 0.240 (0.226, 0.254) |
| **2010** | 3.991 ( 3.933, 4.049 ) | 2.670 (2.623, 2.718 ) | 0.283 (0.268, 0.298) | 0.240 (0.226, 0.254) |
| **2011** | 4.202 ( 4.143, 4.261 ) | 2.825 (2.776, 2.873 ) | 0.314 (0.298, 0.330) | 0.270 (0.255, 0.285) |
| **2012** | 4.491 ( 4.430, 4.551 ) | 2.999 (2.950, 3.049 ) | 0.343 (0.326, 0.359) | 0.296 (0.281, 0.311) |

^1^ In our sensitivity analysis an incident case was defined as the presence of an ICD-9/10 code for HZ plus receipt of antivirals (acyclovir, valacyclovir, famciclovir) within 7 days before or after the diagnostic code for HZ.

^2^ In the sensitivity analysis, the PHN analysis was repeated after 30 days (instead of 90 days) using the same diagnostic criteria.

Table 4. Rate Ratio of Age-Sex Standardized Mean Annual Incidence Rate and 95% Confidence Interval with Bonferroni Correction for Multiple Comparison

|  | **Rate Ratio and 95% Confidence Interval** | | |
| --- | --- | --- | --- |
|  | **Privately Funded (1) vs. Pre-licensure (2)** | **Publicly Funded (1) vs. Pre-licensure (2)** | **Publicly Funded (1) vs. Privately Funded (2)** |
| **HZ** | 1.022 (1.003, 1.040) | 1.218 (1.199, 1.238) | 1.193 (1.180, 1.205) |
| **HZ with Antiviral** | 1.148 (1.121, 1.175) | 1.532 (1.501, 1.563) | 1.335 (1.317, 1.353) |
| **PHN within 90days** | 1.332 (1.226, 1.448) | 2.136 (2.003, 2.278) | 1.603 (1.536, 1.673) |
| **PHN within 30days** | 1.355 (1.237, 1.484) | 2.228 (2.078, 2.389) | 1.645 (1.570, 1.723) |

Table 5: Rate Ratio on Herpes Zoster Incidence and 95% Confidence Interval Using a Regression Model

|  | **Rate Ratio*** | **95% Confidence Interval** | **p-value** |
| --- | --- | --- | --- |
| Non-Publicly vs Publicly Funded Period | 0.86 | (0.44, 1.68) | 0.654 |
| Overall Yearly Trend | 0.99 | (0.90, 1.10) | 0.863 |
| Yearly Trend during Publicly Funded Period | 0.98 | (0.84, 1.13) | 0.750 |
| Age: 0-9 vs 65+ | 1.46 | (0.90, 2.35) | 0.126 |
| Age: 10-44 vs 65+ | 0.22 | (0.14, 0.35) | <0.001 |
| Age: 45-64 vs 65+ | 0.50 | (0.32, 0.79) | 0.003 |
| Female vs Male | 1.16 | (0.83, 1.61) | 0.393 |

*Reference groups: publicly funded period, age 65+, Male
